# Prevalence, cognitive characteristics, and influencing factors of amnestic mild cognitive impairment among older adults residing in an urban community in Chengdu, China

**DOI:** 10.3389/fneur.2024.1336385

**Published:** 2024-01-31

**Authors:** Shan Rao, Yan Cai, Zhujun Zhong, Tianyuan Gou, Yangyang Wang, Shiyi Liao, Peiyuan Qiu, Weihong Kuang

**Affiliations:** ^1^Department of Psychiatry, West China Hospital, Sichuan University, Chengdu, Sichuan, China; ^2^Chengdu Jinxin Mental Diseases Hospital, Chengdu, Sichuan, China; ^3^Evidence-Based Nursing Center, West China Hospital, Sichuan University, Chengdu, Sichuan, China; ^4^Department of Epidemiology and Statistics, West China School of Public Health, Sichuan University, Chengdu, Sichuan, China

**Keywords:** Chinese, amnestic mild cognitive impairment, prevalence, neurocognitive function, older adults

## Abstract

**Objective:**

Dementia is a significant public health concern, and mild cognitive impairment (MCI) serves as a transitional stage between normal aging and dementia. Among the various types of MCI, amnestic MCI (aMCI) has been identified as having a higher likelihood of progressing to Alzheimer’s dimension. However, limited research has been conducted on the prevalence of aMCI in China. Therefore, the objective of this study is to investigate the prevalence of aMCI, examine its cognitive characteristics, and identify associated risk factors.

**Methods:**

In this cross-sectional study, we investigated a sample of 368 older adults aged 60 years and above in the urban communities of Chengdu, China. The participants underwent a battery of neuropsychological assessments, including the Mini-Mental State Examination (MMSE), the Clinical Dementia Rating (CDR), Auditory Verbal Learning Test (AVLT), Wechsler’s Logical Memory Task (LMT), Boston Naming Test (BNT) and Trail Making Test Part A (TMT-A). Social information was collected by standard questionnaire. Multiple logistic regression analysis was utilized to screen for the risk and protective factors of aMCI.

**Results:**

The data analysis included 309 subjects with normal cognitive function and 59 with aMCI, resulting in a prevalence of 16.0% for aMCI. The average age of participants was 69.06 ± 7.30 years, with 56.0% being females. After controlling for age, gender and education, the Spearman partial correlation coefficient between various cognitive assessments and aMCI ranged from −0.52 for the long-term delayed recall scores in AVLT to 0.19 for the time-usage scores in TMT-A. The results indicated that all cognitive domains, except for naming scores (after semantic cue of BNT) and error quantity (in TMT-A), showed statistically significant associations with aMCI. Furthermore, the multiple logistic regression analysis revealed that older age (OR = 1.044, 95%CI: 1.002~1.087), lower educational level, and diabetes (OR = 2.450, 95%CI: 1.246~4.818) were risk factors of aMCI.

**Conclusion:**

This study found a high prevalence of aMCI among older adults in Chengdu, China. Individuals with aMCI exhibited lower cognitive function in memory, language, and executive domains, with long-term delayed recall showing the strongest association. Clinicians should prioritize individuals with verbal learning and memory difficulties, especially long-term delayed recall, in clinical practice.

## Introduction

Alzheimer’s Disease (AD) poses a significant public health challenge, resulting in huge disease burdens for families, health-care systems, and societies worldwide. It is estimated that approximately 40–50 million people are currently living with dementia, and this number is projected to reach 131.5 million by 2050 due to the aging global populations (1, 2). Low- and middle-income countries bear a considerable burden of dementia, and China, as the most populous middle-income nation, accounts for a significant proportion of individuals diagnosed with dementia.

Given the absence of a cure for AD, early intervention measures are recognized as the most cost-effective approach to manage the disease (3, 4). Research suggests that delaying the onset of AD dementia by just 5 years could lead to a 57% reduction in AD cases and save 344 billion to 627 billion US dollars (5). Therefore, there is a crucial need to shift the focus of AD diagnosis and treatment to earlier stages.

Mild cognitive impairment (MCI) is considered an intermediate phase between normal cognitive aging and overt dementia. Individual with MCI has objective impairment in one or more cognitive domains that differ from healthy age-matched individuals, accompanied by normal general cognitive function and relatively intact activities of daily living (6, 7). As an early stage of dementia, MCI is believed by some researchers to have an annual progression rate to clinically diagnosed dementia ranging from 7% to 22% (6–12). It is important to note that not all individuals with MCI will develop AD dementia, emphasizing the importance of studying clinical subtypes of MCI. Based on the involvement of memory, MCI can be classified into two subtypes: amnestic MCI (aMCI), where memory impairment is predominant, and non-amnestic MCI (naMCI), where memory is unaffected (13). Recent evidence indicates that individuals with aMCI have approximately twice the likelihood of developing AD compared to those with naMCI (8, 14, 15).

To gain a better understand the prevalence of aMCI and associated risk factors, it is essential to examine findings from different cultures and populations. In the United States, Michau’s study reported an incidence of 12.4% for aMCI using data from the Uniform Data Set (UDS) of the National Alzheimer’s Coordinating Center (NACC) (9). In Australia, Pusswald found that the incidence was 15.4% for aMCI in a memory outpatient clinic (16). A Swedish study by Overton showed that the prevalence of aMCI was 7.34% among older adults aged 60 years old and above (17). The reported prevalence of aMCI in China varied, ranging from 10.9% to 17.1% (18–21). Furthermore, researchers have also made efforts to identify associated factors for aMCI, with age and education emerging as important risk factors (22–24). However, the results regarding other potential associated factors including hypertension, diabetes, sleep disturbance, physical exercise, smoking, and drinking, have not shown consistent associations (23, 25, 26).

To address the inconsistencies and regional differences in previous research, particularly regarding the prevalence and risk factors of aMCI, our study aimed to investigate these aspects among urban community-dwelling older adults aged 60 years and above in Chengdu, Sichuan Province. Additionally, we aimed to explore the cognitive characteristics of individuals with aMCI, an area that has been underexplored. Notably, no relevant studies have been conducted in this specific location. By comparing the prevalence of aMCI, examining its cognitive characteristics, and identifying associated risk factors in different cultures, including our study in Chengdu, we aim to contribute valuable insights to the existing knowledge in this field and provide a comprehensive understanding of aMCI within our specific study population.

## Methods

### Study design and participants

A sample of urban older adults was obtained from a cross-sectional survey. The survey was conducted in Jinjiang District, one of the 12 districts in Chengdu, Sichuan Province in China. Chengdu is an important central city in western China, ranking eighth in Gross Domestic Product (GDP) among all cities in China, generating 1,217.02 billion yuan in 2016. Jinjiang District also belongs to one of the central districts of Chengdu, ranking fifth in GDP (among all districts in China) with 83.46 billion yuan in 2016. In this area, 21.62% of people were aged 60 years and over in 2016. A multi-stage cluster sampling method was applied to ensure that study participants were selected from various socio-economic sectors, which made it more representative of the population. The 11 towns in Jinjiang District were divided into three levels based on income level – low, middle, and high. Six residential areas were selected randomly from each group, with a total of 18 residential areas. Then we randomly selected three to six buildings from each residential area and investigated all older adults meeting the inclusion criteria. The data was collected from October 2016 to March 2017.

The inclusion criteria for participants were as below: (1) individuals who were permanent residents (residing for a duration of at least 12 months); and (2) Aged 60 years and older. Participants were excluded if they: (1) Experienced severe visual or hearing impairments, serious physical illness, or weakness that hindered their ability to complete the survey; (2) Had a history of traumatic brain injuries or psychiatric disorders that could impact cognitive function; (3) Exhibited symptoms of depressive disorders, defined as a Chinese self-reported version of the geriatric depression inventory (GDI-SR) score ≥ 3 (27); (4) Were classified as demented, defined as having a Clinical Dementia Rating (CDR) score ≥ 1 or adjusted MMSE scores based on educational level (≤17 for illiterate, ≤20 for primary school and ≤24 for above the middle school); and (5) had naMCI, defined as objective non-memory impairment according to a z score ≤ −1.5 for at least one non-memory neuropsychological test, a CDR score of 0.5, preserved general cognitive function according to MMSE scores adjusted by educational level (>17 for illiterate, >20 for primary school, and >24 for above the middle school), intact daily living ability and absence of dementia. In total, 617 randomly selected older adults participated this survey. Among these, 13 participants had visual or hearing impairments, 20 refused to answer questions, 84 participants exhibited symptoms of depression, 39 participants gave incomplete data, 91 participants were potentially diagnosed with dementia, and 2 participants had naMCI. Ultimately, a total of 368 older adults were included for analysis.

The survey protocol (including the informed consent) was approved by the Medical Ethics Committee of Sichuan University. All the participants signed the informed consent forms. Qualified research assistants with medical backgrounds and community physicians administered this survey. All the research assistants and clinicians were intensively trained by psychiatrists from the Department of Psychiatry of West China Hospital.

### Cognitive assessment

All participants underwent the following neuropsychological assessments: Mini-Mental State Examination (MMSE), the Clinical Dementia Rating (CDR), Auditory Verbal Learning Test (AVLT), Wechsler’s Logical Memory Task (LMT), Boston Naming Test (BNT) and Trail Making Test Part A (TMT-A).

The MMSE scale was used to assess global cognitive function (28). The MMSE consists of multiple questions and covers six cognitive domains: orientation (10 points), immediate memory (3 points), attention and calculation (5 points), recall ability (3 points), language (8 points), and visuospatial ability (1 point). Usually, the visuospatial ability task was classified as one of the language items. The score totals ranged from 0 to 30, with higher scores indicating better cognitive function.

The CDR is a reliable tool for staging dementia severity (29). It includes six cognitive categories, namely memory, orientation, judgment, and problem-solving, community affairs, home and hobbies, and personal care. According to clinical scoring rules, CDR 0 indicates no dementia, CDR 0.5 indicates questionable dementia, CDR 1 indicates mild dementia, CDR 2 indicates moderate dementia and CDR 3 indicates severe dementia. In our study, the CDR was also used to assess cognitive complaints and activities of daily living. Trained psychiatrists from the Department of Psychiatry of West China Hospital were responsible for conducting the CDR ratings.

The AVLT is a well-recognized measure used to assess verbal learning and memory (30). In this test, the examiner read a list of 12 unrelated Chinese words three times. Immediately following each presentation and after 20 min delay, participants were required to recall as many words as possible without a time constraints and in any order. The immediate recall scores consisted of the number of words recalled in each trial (ranging from 0 to 12) and the total number of words recalled across the three immediate trials (ranging from 0 to 36). The delayed score consisted of the number of words recalled after the 20-min delay (ranging from 0 to 12), which we refer to as the long-term delayed recall in our study. Finally, the participants were presented with the word list.

LMT primarily tested participants’ logical memory (31). The participants were told a short story orally, which contained 20 underlining keywords. Then the examinee was asked to recall the story (immediate recall). Approximately 20 or 30 min later, free recall of the story was again elicited (delayed recall).

BNT was used to assess language ability (32). Participants were presented with 30 images and asked to provide the corresponding names. For correct responses, including self-corrections, credits were awarded as “spontaneous naming scores (SN).” If a participant gave a wrong response or gave no response within 20 s, the examiner provided a standard semantic cue. If a participant was able to provide the correct answer with the cue, credit was given and recorded as “naming scores after phonemic cue (CN).”

TMT-A was used to assess the execution function of participants (33). It required the participants to link numbers from 1 to 25 as fast as possible while keeping the nib on the page. The amount of time consumed, and the number of errors made were recorded, defined as TMT-A (s) and TMT-A error, respectively.

### Social information

Social information was obtained through participants or their appropriate informants. The following data was collected by standard questionnaire: (1) Demographic data such as age, gender, educational level, marital status, and income (average monthly income per person in family); (2) History of chronic diseases including hypertension, diabetes, coronary heart disease and cerebrovascular disease; (3) Daily living information (sleep disorders, smoking and drinking alcohol). Specifically, marital status consisted of two categories: “marriage” and “no marriage.” The latter was defined as being divorced, widowed, or unmarried.

The sleep disorders of older adults were assessed by the Chinese version of Pittsburgh Sleep Quality Index scale (PSQI) (34). The PSQI assessed sleep quality over the past month and contains of 19 items scored on a 3-point Likert scale. It encompasses seven domains, including subjective sleep quality, sleep latency, sleep duration, sleep efficiency, sleep disturbances, use of sleep medications and daytime dysfunction. The global PSQI scores ranged from 0 to 21, with higher total scores indicating poorer sleep quality. The Chinese version of the PSQI has been shown to possess good reliability and validity (35).

### Diagnostic criteria

Diagnosis of aMCI was made based on Petersen’s criteria (36–38): (1) Memory complaint by participants, preferably corroborated by their informants; (2) Objective memory impairment according to a z score ≤ −1.5 for at least one memory neuropsychological test and a CDR score of 0.5; (3) Preserved general cognitive function according to MMSE scores adjusted by educational level (39) (>17 for illiterate, >20 for primary school and >24 for above the middle school); (4) Intact daily living ability; and (5) Absence of dementia. Ultimately, among 368 older adults in this study, 59 were aMCI participants and 309 were participants with normal cognitive function.

### Statistical analysis

The continuous variable was presented as mean ± standard deviation (SD) and categorical variables were described in terms of frequency (%). The Spearman correlation coefficient and the Spearman partial correlation coefficient controlling age, gender and educational level were used to detect differences in cognitive test results between the aMCI group and normal group (individuals in the aMCI group were assigned a value of 1 and individuals in the normal group were assigned a value of 0). A comparison of continuous data among aMCI participants and those with normal cognitive function was performed using independent-sample *t*-test analysis. Chi-square tests were applied to examine group differences in dichotomous variables data. For the ordinal categorical variable (educational level), the Cochran-Armitage test for trend was used to verify whether the prevalence of aMCI is higher with the lower educational level. Multiple logistic regression analysis was utilized to screen for the risk and protective factors of aMCI. The reported *p* values are the results of two-sided tests. *p* values of <0.05 were considered as statistically significant. Statistical analysis was performed using Stata version 15.1.

## Results

### Prevalence and cognitive characteristics

The prevalence of aMCI in this study was found to be 16.0%. Table 1 provides a summary of the cognitive characteristics of participants with aMCI compared to those with normal cognitive function. The unadjusted Spearman correlation coefficients ranged from −0.58 for AVLT-LR to 0.26 for TMT-A (s). Results revealed that participants with aMCI had lower scores on MMSE, AVLT, LMT, and spontaneous naming scores (BNT) compared to cognitively normal participants. Additionally, individuals with aMCI demonstrated longer completion times and more errors in TMT-A compared to the normal group. After controlling for age, gender and educational level, the Spearman partial correlation coefficient ranged from −0.52 for the AVLT-LR to 0.19 for the TMT-A (s). Participants in the aMCI group continue to perform worse on most cognitive assessments compared to the normal group. While there were no significant differences between the groups in naming scores after semantic cue of BNT based on unadjusted or adjusted correlational analysis, we also did not see significant differences in TMT-A errors after adjusting for age, gender, and education. By using a z score ≤ −1.5 for each neuropsychological test, we identified that among participants with aMCI, 29 (49.2%) had memory impairment only, 13 (22.0%) had memory and language (BNT) impairments, 12 (20.3%) had memory and executive function (TMT-A) impairments, and 5 (8.5%) had memory, language (BNT) and executive function (TMT-A) impairments.

**Table 1:** Cognitive assessment scores in participants with aMCI and normal group.

Cognitive assessments	Total (*n* = 368)	aMCI (*n* = 59)	Normal (*n* = 309)	Spearman *ρ*	*p*	Spearman ρ^*^	*p*
MMSE	26.56 ± 2.47	24.17 ± 2.98	27.02 ± 2.07	−0.36	<0.001	−0.29	<0.001
**AVLT**							
AVLT-1	2.93 ± 1.66	1.85 ± 1.14	3.14 ± 1.66	−0.28	<0.001	−0.21	<0.001
AVLT-2	5.12 ± 2.16	3.12 ± 1.49	5.50 ± 2.05	−0.41	<0.001	−0.33	<0.001
AVLT-3	6.28 ± 2.67	3.66 ± 1.61	6.78 ± 2.54	−0.44	<0.001	−0.37	<0.001
AVLT-sum 1–3	14.33 ± 5.66	8.63 ± 3.20	15.42 ± 5.36	−0.46	<0.001	−0.37	<0.001
AVLT-LR	5.06 ± 2.92	1.19 ± 0.92	5.80 ± 2.56	−0.58	<0.001	−0.52	<0.001
**LMT**							
LMT-IR	5.14 ± 3.22	2.97 ± 2.89	5.55 ± 3.11	−0.31	<0.001	−0.19	<0.001
LMT-DR	4.52 ± 3.28	2.49 ± 3.09	4.90 ± 3.18	−0.29	<0.001	−0.19	<0.001
**BNT**							
BNT-SN	19.47 ± 4.35	16.02 ± 4.54	20.13 ± 3.99	−0.32	<0.001	−0.21	<0.001
BNT-CN	1.60 ± 2.10	1.64 ± 1.64	1.59 ± 2.18	0.03	0.555	−0.05	0.310
**TMT-A**							
TMT-A (s)	78.46 ± 41.38	107.75 ± 57.35	72.86 ± 35.00	0.26	<0.001	0.19	<0.001
TMT-A error	0.92 ± 2.26	1.68 ± 2.82	0.78 ± 2.11	0.19	<0.001	0.08	0.151

AVLT-LR, long-term delayed recall scores of Auditory Verbal Learning Test; LMT-IR, immediate recall scores of Logical Memory Test; LMT-DR, delayed recall scores of Logical Memory Test; BNT-SN, spontaneous naming scores of Boston Naming Test; BNT-CN, naming scores after semantic cue of Boston Naming Test. ^*^Spearman partial correlation coefficient for the association between aMCI and normal group (the aMCI group was assigned a value of 1, and the normal group was assigned a value of 0), controlled for age, gender, and educational level.

### Sociodemographic and clinical characteristics

In this study, the average age of participants was 69.06 ± 7.30 years old. Female participants accounted for 56.0%, and participants with a middle school education and above accounted for 60.3%. The participants’ sociodemographic and clinical characteristics were presented in Table 2. The age, prevalence of hypertension and diabetes between participants with aMCI, and normal group were statistically different. And the results of Cochran-Armitage test for trend indicated that the prevalence of aMCI was increased with decreasing educational level. However, participants with aMCI and participants in the normal group did not differ in gender, marital status, income, coronary heart disease, cerebrovascular disease, sleep disorders, smoking, or drinking.

**Table 2:** Comparison of sociodemographic and clinical characteristics between participants with aMCI and normal group.

Variables	Total (*n* = 368)	aMCI (*n* = 59)	Normal (*n* = 309)	*t*/*χ^2^*	Value of *p*
**Demographic factors**					
Age (Mean ± SD)	69.06 ± 7.30	72.97 ± 9.06	68.32 ± 6.68	−4.60	<0.001
Gender (%)				0.32	0.572
Male	162 (44.0)	24 (14.8)	138 (85.2)		
Female	206 (56.0)	35 (17.0)	171 (83.0)		
Education (%)				40.96	<0.001
Above middle school	222 (60.3)	16 (7.2)	206 (92.8)		
Primary school	102 (27.7)	24 (23.5)	78 (76.5)		
Illiteracy	44 (12.0)	19 (43.2)	25 (56.8)		
Marital status (%)				2.46	0.117
Marriage	301 (81.8)	44 (14.6)	257 (85.4)		
No marriage	67 (18.2)	15 (22.4)	52 (77.6)		
Income (%)				0.02	0.882
>3,000 yuan	109 (29.6)	17 (15.6)	92 (84.4)		
≤3,000 yuan	259 (70.4)	42 (16.2)	217 (83.8)		
**Chronic diseases**					
Hypertension (%)				8.54	0.003
No	195 (53.0)	21 (10.8)	174 (89.2)		
Yes	173 (47.0)	38 (22.0)	135 (78.0)		
Diabetes (%)				10.43	0.001
No	289 (78.5)	37 (12.8)	252 (87.2)		
Yes	79 (21.5)	22 (27.9)	57 (72.1)		
Coronary heart disease (%)				0.18	0.672
No	330 (89.7)	52 (15.8)	278 (84.2)		
Yes	38 (10.3)	7 (18.4)	31 (81.6)		
Cerebrovascular disease (%)				2.38	0.123
No	332 (90.2)	50 (15.1)	282 (84.9)		
Yes	36 (9.8)	9 (25.0)	27 (75.0)		
**Daily living information**					
Sleep disorders (Mean ± SD)	6.07 ± 4.23	6.41 ± 4.45	6.01 ± 4.19	−0.66	0.509
Smoking (%)				2.55	0.110
No	304 (82.6)	53 (17.4)	251 (82.6)		
Yes	64 (17.4)	6 (9.4)	58 (90.6)		
Drinking (%)				1.81	0.178
No	141 (38.3)	18 (12.8)	123 (87.2)		
Yes	227 (61.7)	41 (18.1)	186 (81.9)		

SD, standard deviation.

### Risk factors

In the multiple logistic regression analysis, our model included sociodemographic and clinical characteristics that were statistically associated with the prevalence of aMCI. As such, age, educational level, hypertension, and diabetes were selected. Considered as a confounder, gender was also placed in the model. The results presented in Table 3 revealed that older age (*OR* = 1.044, 95%*CI*: 1.002~1.087), obtaining less education, and having diabetes (*OR* = 2.450, 95%*CI*: 1.246~4.818) were risk factors of aMCI. Particularly, the risk of aMCI was nearly twice as high among illiterate individuals (*OR* = 8.161, 95%*CI*: 3.402~19.575) compared to those with primary school education (*OR* = 3.746, 95%*CI*: 1.816~7.724), using participants with middle school education or above as the reference. However, no statistically significant association was observed between gender and hypertension, and the prevalence of aMCI.

**Table 3:** Multiple logistic regression analysis of risk factors for aMCI.

Variables	*B*	*SE*	*Wald* score	Value of *p*	*OR*	95%*CI*
Age	0.043	0.021	2.040	0.042	1.044	1.002 ~ 1.087
Gender						
Male	—	—	—	—	—	1.000
Female	−0.252	0.330	−0.760	0.445	0.777	0.407 ~ 1.485
Education level						
Above middle school	—	—	—	—	—	1.000
Primary school	1.321	0.369	3.580	<0.001	3.746	1.816 ~ 7.724
Illiteracy	2.099	0.446	4.700	<0.001	8.161	3.402 ~ 19.575
Hypertension						
No	—	—	—	—	—	1.000
Yes	0.573	0.324	1.770	0.077	1.773	0.940 ~ 3.343
Diabetes						
No	—	—	—	—	—	1.000
Yes	0.896	0.345	2.600	0.009	2.450	1.246 ~ 4.818

SE, Standard Error; OR, Odds Ratio; CI, Confidence Interval.

## Discussion

To the best of our knowledge, this is the first study conducted in Western China that specifically focuses on the prevalence and characteristics of aMCI and its associated factors among older adults aged 60 years and above in the urban communities. Our study contributes to the existing literature by providing insights into the prevalence of aMCI in Chengdu. In our study, we found that the prevalence of aMCI among older adults in community-dwellings in Chengdu was 16.0%. This prevalence is consistent with the findings of previous studies conducted by Li (17.1% prevalence of aMCI among older adults aged 60 years old and above) (19), and Zhang (16.1% prevalence of aMCI among older adults in Shijiazhuang) (21) in China. It is worth noting that the reported prevalence rates of aMCI in many other countries have been found to be lower, ranging between 7.3% and 11.6% (17, 24, 40, 41).

On the one hand, the observed differences between countries, including our study, could be attributed to various factors such as differences in survey scales, sample size, and sampling methods employed across different cultural contexts. These methodological variations can influence the prevalence rates and make direct comparisons challenging. On the other hand, early life experiences may impact on cognitive health in later in life. Our participants were born before 1957, which means they may have experienced significant historical events that could have influenced their cognitive health. For instance, they may have lived through the Great Famine in the early 1960s, a period of severe food scarcity and malnutrition during their developmental years. In addition, our participants also experienced the Cultural Revolution, a time characterized by social upheaval in China. During this period, education opportunities was limited, and pursuing formal education was often discouraged. It is widely recognized that education acts as an important protective factor against cognitive decline (42, 43). The limited access to education during the Cultural Revolution may have further contributed to the higher prevalence of aMCI observed in our study population. Further research is needed to explore the long-term effects of early life experiences on cognitive health and to better understand the complex interplay between education and cognitive decline.

After adjusting for age, gender, and education, we observed significant differences in almost all global cognitive functions and individual neuropsychological domains between individuals with normal cognitive function and those with aMCI. However, there were no significant differences in semantic cue naming scores of the Boston Naming Test and the number of errors in Trail Making Test Part A. Among the various cognitive domains, verbal learning and recall showed stronger relationships with aMCI, with delayed recall demonstrating the strongest association. This finding is in line with previous studies. Zhao’s study found that both short-term delay and long-term delay recall were equally effective in identifying aMCI patients (44). Fisher’s and Simon’s study suggested that delayed recall had a higher predictive value for the conversion of aMCI to AD compared to other domains of cognitive test (45, 46).

In our study, we found that increasing age was associated with increased prevalence of aMCI. Age is also recognized as the most important factor for AD, as older age is linked to higher rates of AD. This is evident in various populations, including China and the United State. In a systematic review conducted in China in 2010, the prevalence of AD was found to be 1.27% among individuals aged 65 to 69 years and 18.54% among those aged 85 to 89 years (47). The 2020 Alzheimer’s Disease Facts and Figures reported that in the United States, the percentage of people with AD increases greatly with age: 3% for individuals aged 65–74, 17% for individuals aged 75–84, and 32% for individuals aged 85 or older (48). Similar patterns have been observed worldwide. aMCI, as a subtype of MCI, is characterized by memory impairment and is strongly associated with the development of AD. The relationship between age and prevalence rates of aMCI in different countries has yield mixed results. While many studies have reported that age as a risk factor for aMCI (9, 17, 22), Sosa’s study conducted in Latin America, China, and India demonstrated that although some countries showed positive associations between age and aMCI prevalence, others exhibited negative associations (49). However, it is important to note that age consistently remains a risk factor for the conversion from aMCI/MCI to AD (50). It is worth emphasizing that aMCI is not a normal part of aging, and advanced age alone is not sufficient to cause aMCI.

A second finding was that education was a protective factor for aMCI. This is consistent with previous studies indicating that increasing education is negatively related to aMCI, MCI, AD, and is also protective against the development of AD (20, 51, 52). This could be explained by the Cognitive Reserve Hypothesis. To highlight this point, research has shown that having a higher cognitive reserve, which refers to the brain’s ability to make flexible and efficient use of cognitive networks (networks of neuron-to-neuron connections), helps a person’s ability to cope and compensate for brain damage (48, 53). Therefore, it is reasonable that having a higher education level can delay the progression of cognitive impairment and AD by way of increasing one’s cognitive reserve.

We also identified diabetes as a risk factor for aMCI in our study, which is consistent with previous research. Both cross-sectional and longitudinal studies have demonstrated the influence of diabetes on cognitive impairment (54–56). However, the underlying mechanism through which diabetes impairs cognitive function is still unclear. One theory suggests that cerebral insulin resistance promotes the phosphorylation of tau protein, making the brain more susceptibility to neurodegeneration, potentially leading to AD (57). Another emerging concept in mechanistic studies is the potential role of advanced glycation end products (AGEs). AGEs contribute to the production of reactive oxygen species, which in turn promote oxidative stress and inflammatory cytokines, resulting in diabetes-associated neurovascular damage (58). Furthermore, diabetes has been associated with abnormalities such as accelerated hippocampal atrophy and reduction in whole brain volume, indicating a potential role in neurodegeneration (55).

We did not observe an association between hypertension and aMCI after adjusting for demographic factors. Although some studies have reported an association between hypertension and cognitive impairment and dementia (59, 60), the effects of hypertension on the subtypes of MCI remain unclear. For instance, Casado’s study found an association between hypertension and aMCI (61), while others have reported a relationship with naMCI, particularly in relation to vascular dementia (VaD) (24, 59). Additionally, evidence suggests that aMCI has a greater tendency to progress to AD, while naMCI is more likely to develop into VaD (14, 25). In our study, we did not observe an association between aMCI and cardio-cerebrovascular diseases, smoking, and drinking. This may indicate that these factors are not directly related to aMCI but rather to naMCI. However, we did not have enough naMCI cases in our study to investigate the relationship between hypertension and other cardio-cerebrovascular diseases with naMCI. Furthermore, considering that many current studies do not consider the severity or duration of hypertension, there is an opportunity to expand our understanding of how hypertension and cardio-cerebrovascular diseases impact aMCI, naMCI, VaD, and AD. This could be further explored with a well-designed cohort study.

Finally, we did not find an association between aMCI and sleep disorders. While several studies have shown that sleep disturbances increase risk of aMCI (62–64), there are also studies have demonstrated the opposite findings. For example, Gavuoto’s study conducted among a large sample of community-dwelling older adults found that those with less sleep disruption were more like to report higher levels of subjective memory decline, which is one of the diagnostic criteria for aMCI (65). The authors argued that this might be attributed to compensatory sleep behavior in response to increased cognitive effort to compensatory sleep behavior in response to increased cognitive effort to maintain memory function (65). It should be noted that the Pittsburgh Sleep Quality Index scale used in our study does not include measures of sleep depth and stages, which indicate different sleep patterns. Analyzing sleep patterns would provide valuable insights into which specific sleep disturbances are associated with aMCI. A system review found that individuals with aMCI may experience more disturbances in sleep efficiency and slow-wave sleep (66). Therefore, further longitudinal analyses of cohorts will be necessary to fully evaluate the associations between sleep and aMCI.

Our study provides novel insights into the prevalence and characteristics of aMCI in Chengdu, shedding light on potential regional variations in its prevalence compared to other countries. Understanding these epidemiological differences is crucial for developing targeted interventions and healthcare strategies in the region. Additionally, our findings highlight the differential relationships between various cognitive domains and aMCI, emphasizing the importance of assessing verbal learning and recall, specifically delayed recall. Clinicians should prioritize patients with difficulties in these areas, particularly long-term delayed recall, in clinical practice.

We acknowledge several important limitations as well. Firstly, it is important to note that our sample is representative of urban settings, which may differ significantly from rural settings in terms of economic factors, culture influences, and other relevant variables. Therefore, caution should be exercised when generalizing our results to older adults residing in rural areas. Additionally, it is important to recognize that community samples may exhibit milder symptoms compared to clinical samples. As a result, our findings may not be applicable to individuals diagnosed with clinical aMCI. Secondly, it is crucial to highlight that our study design was a cross-sectional in nature. Therefore, it is not appropriate to infer causal relationships. To gain a deeper understanding of the dynamic processes that potentially contribute to the development of aMCI, further research utilizing longitudinal designs is warranted.

## Conclusion

This study revealed a high prevalence of aMCI among urban, community-dwelling older adults aged 60 years and over in Chengdu, China. Compared with cognitively normal persons, individuals with aMCI exhibited lower cognitive function in memory, language, and executive domains. Notably, long-term delayed recall demonstrated the strongest association with aMCI, even after adjusting age, gender, and education. These findings suggest that clinicians should prioritize individuals with verbal learning and memory difficulties, particularly those with challenges in long-term delayed recall, in clinical practice. Furthermore, this study identified older age, lower education, and diabetes as factors associated with aMCI. These results highlight the importance of cognitive training and effective management of chronic disease in preventing and delaying the onset of Alzheimer’s dementia.

## Data availability statement

The raw data supporting the conclusions of this article will be made available by the authors, without undue reservation.

## Ethics statement

The studies involving humans were approved by Special Committee on Clinical trial and Biomedical Ethics in West China Hospital of Sichuan University. The studies were conducted in accordance with the local legislation and institutional requirements. Written informed consent for participation in this study was provided by the participants' legal guardians/next of kin.

## Author contributions

SR: Investigation, Writing – original draft. YC: Writing – original draft, Formal analysis. ZZ: Investigation, Writing – review & editing. TG: Investigation, Writing – review & editing. YW: Writing – review & editing, Formal analysis. SL: Formal analysis, Writing – review & editing. PQ: Writing – review & editing, Conceptualization, Methodology, Resources. WK: Conceptualization, Methodology, Resources, Writing – review & editing.

## Figures and Tables

**Table:** 

MCI	Mild cognitive impairment
aMCI	Amnestic MCI
MMSE	Mini-Mental State Examination
CDR	Clinical Dementia Rating
AVLT	Auditory Verbal Learning Test
LMT	Wechsler’s Logical Memory Task
BNT	Boston Naming Test
TMT-A	Trail Making Test Part A
AD	Alzheimer’s Disease
naMCI	Non-amnestic MCI
UDS	Uniform Data Set
NACC	National Alzheimer’s Coordinating Center
GDP	Gross Domestic Product
GDI-SR	Self-reported version geriatric depression inventory
SN	Spontaneous naming scores
CN	Naming scores after phonemic cue
PSQI	Sleep Quality Index scale
SD	Standard deviation
DMN	Default mode network
PCC	Posterior cingulate cortex
MPFC	Medial prefrontal cortex
AGEs	Advanced glycation end products
